# Supplementary material for: GABAA Receptor β3 Subunit Mutation N328D Heterozygous Knock-in Mice Have Lennox–Gastaut Syndrome
Source: Int J Mol Sci. 2023 May 8;24(9):8458. doi: 10.3390/ijms24098458 (PMC10179596; doi:10.3390/ijms24098458)
Supplement: Supplementary file 1 [file ijms-24-08458-s001.zip › ijms-2316772-supplementary.pdf]

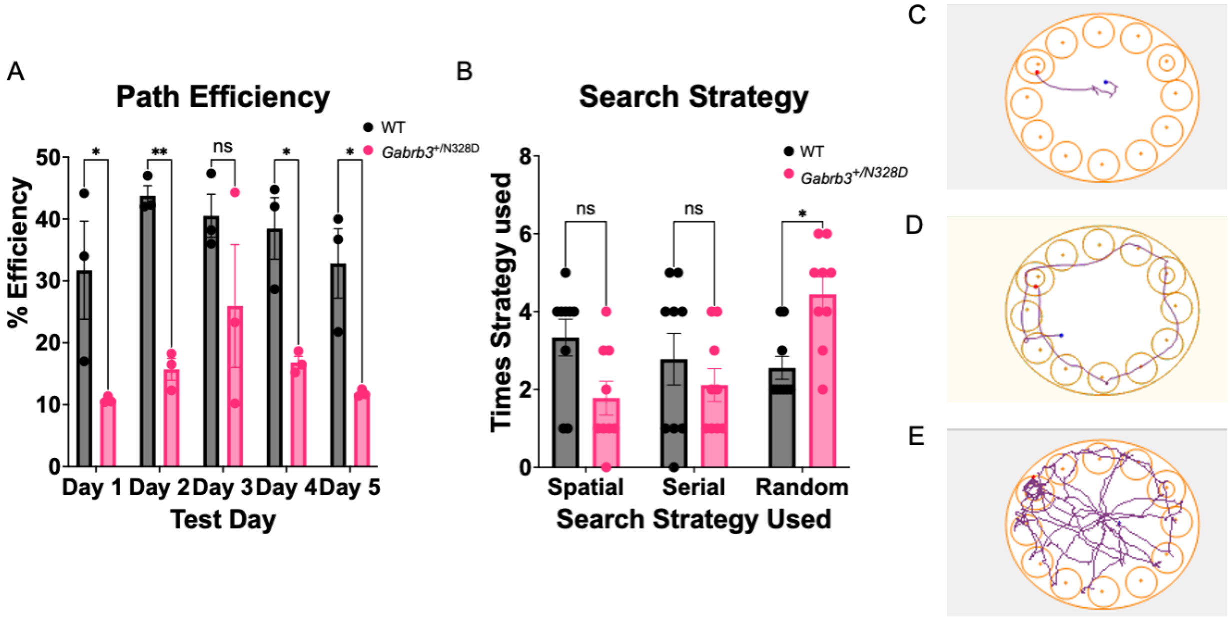

Supplemental Figure S1. Gabrb3$^{+/N328D}$ mice show reduced efficiency and a more random search pattern. (A) The path efficiency percentage of *Gabrb3$^{+/N328D}$* and wild-type littermates was observed showing a significant reduction across four out of five training days. (B) The search strategy used by each mouse was assessed comparing spatial (C), serial (D), and random (E) search strategies. *Gabrb3$^{+/N328D}$* mice displayed the random search strategy significantly more than wild-type littermates. ** $p < 0.05$, ** $p < 0.01$ by two-way ANOVA with post hoc Sidak's multiple comparison's test.*

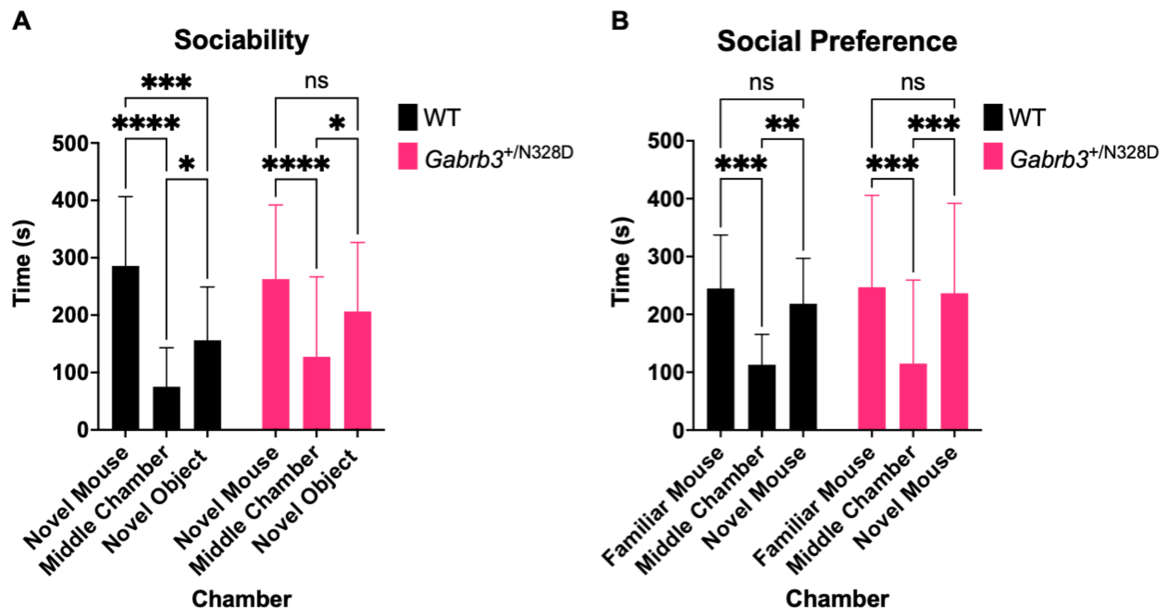

Supplementary Figure S2. Gabrb3$^{+/N328D}$ mice did not show a significant sociability or social preference deficit. (A) Sociability was assessed in *Gabrb3*$^{+/N328D}$ mice. Mutant mice spent significantly more time with the novel mouse or novel object than in the middle chamber, and there was not a significant difference between time spent between novel mouse and novel object. (B) *Gabrb3*$^{+/N328D}$ mice did not show a significant social preference deficit. Mice did not spend a significantly different amount of time between the familiar and novel mice. ** $p < 0.05$, ** $p < 0.01$, *** $p \leq 0.01$ and **** $p < 0.0001$ by two-way ANOVA with post hoc Sidak's multiple comparison's test*

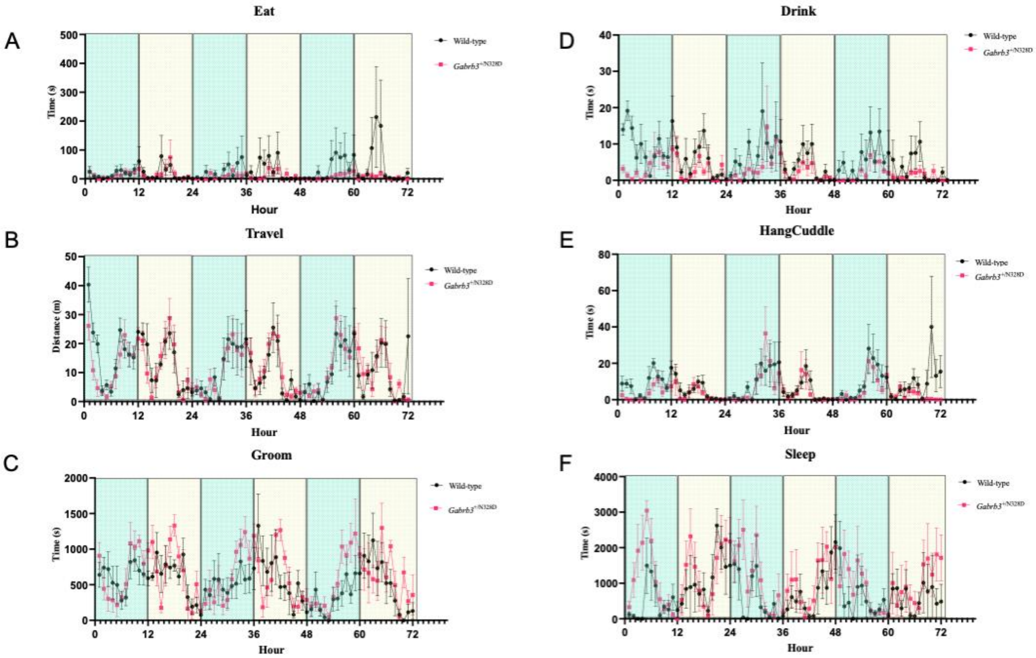

Supplementary Figure S3. Gabrb3$^{+/N328D}$ mice show a significant decrease in drinking and increased sleeping. (A-C) Mutant mice did not show a significant difference in eating, travelling, or grooming behavior during home cage monitoring. (D) Drinking was seen decreased in mutant mice and the amount of time spent sleeping (F) showed an increase in *Gabrb3*$^{+/N328D}$ mice. There was not a significant difference in hang cuddle behavior (E).
